# Distribution, scale, and drivers of mass mortality events in Europe's freshwater bivalves

**DOI:** 10.1111/cobi.70192

**Published:** 2025-12-18

**Authors:** Daniel A. Cossey, Maria Urbańska, Ronaldo Sousa, Juergen Geist, Anna Maria Labecka, Şebnem Atasaral, Maciej Bonk, Liliya Bylyna, Frank P. L. Collas, Daniel Daill, Andreas H. Dobler, Noé Ferreira‐Rodríguez, Dariusz Halabowski, Martina I. Ilarri, Jasna Lajtner, Jon H. Mageroy, Evelyn A. Moorkens, Javier Morales, William H. Morgan, Grégory Motte, Keiko Nakamura, Paz Ondina, Martin Österling, Małgorzata Ożgo, Momir Paunović, Vincent Prié, Maja Raković, Larysa Shevchuk, Spase Shumka, Mikhail O. Son, Jouni Taskinen, Frankie Thielen, Henn Timm, Jelena Tomović, Dariusz Ulikowski, Gorazd Urbanič, Simone Varandas, Agnieszka Izolda Wasilewska, Niklas Wengström, David C. Aldridge

**Affiliations:** ^1^ Department of Zoology University of Cambridge Cambridge UK; ^2^ Department of Zoology Poznań University of Life Sciences Poznań Poland; ^3^ CBMA – Centre for Molecular and Environmental Biology/ARNET‐Aquatic Research Network/ IB‐S, Institute of Science and Innovation for Bio‐Sustainability, Department of Biology University of Minho, Campus Gualtar Braga Portugal; ^4^ Aquatic Systems Biology Unit, School of Life Sciences Technical University of Munich Freising Germany; ^5^ Faculty of Biology, Institute of Environmental Sciences, Life History Evolution Group Jagiellonian University Kraków Poland; ^6^ Department of Fisheries Technology Engineering, Faculty of Marine Sciences Karadeniz Technical University Trabzon Türkiye; ^7^ Institute of Nature Conservation Polish Academy of Sciences Kraków Poland; ^8^ Berdychiv Medical College Berdychiv Ukraine; ^9^ Department of Environmental Science, Radboud Institute for Biological and Environmental Science (RIBES) Radboud University Nijmegen The Netherlands; ^10^ Consultants in Aquatic Ecology and Engineering ‐ blattfisch e.U. Wels Austria; ^11^ Faculty of Biology and Environmental Protection, Department of Ecology and Vertebrate Zoology University of Lodz Lodz Poland; ^12^ Interdisciplinary Centre of Marine and Environmental Research (CIIMAR/CIMAR) University of Porto Matosinhos Portugal; ^13^ Department of Biology, Faculty of Science University of Zagreb Zagreb Croatia; ^14^ Norwegian Institute for Nature Research Oslo Norway; ^15^ Department of Zoology, Trinity College University of Dublin Dublin Ireland; ^16^ Department of Zoology University of Salamanca Salamanca Spain; ^17^ Public Service of Wallonia, Agriculture, Natural Resources and the Environment DEMNA, Directorate of Nature and Water Gembloux Belgium; ^18^ Environmental Service Department Sociedad Aragonesa de Gestión Agroambiental (SARGA) Zaragoza Spain; ^19^ Department of Zooloxía, Xenética e Antropoloxía Física, Facultade de Veterinaria, Instituto de Biodiversidade Agraria e Desenvolvemento Rural (IBADER), Universidade de Santiago de Compostela Lugo Spain; ^20^ River Ecology and Management, Research Group RivEM, Department of Environmental and Life Sciences Karlstad University Karlstad Sweden; ^21^ Department of Evolutionary Biology Kazimierz Wielki University Bydgoszcz Poland; ^22^ Department of Hydroecology and Water Protection, Institute for Biological Research “Siniša Stanković” National Institute of Republic of Serbia University of Belgrade Belgrade Serbia; ^23^ Institut Systématique Evolution Biodiversité (ISYEB), Muséum national d'Histoire naturelle, CNRS, Sorbonne Université, EPHE Université des Antilles Paris France; ^24^ Department of Earth Sciences Zhytomyr Polytechnic State University Zhytomyr Ukraine; ^25^ Department of Food and Biotechnology, Faculty of Biotechnology and Food Agricultural University of Tirana Tirana Albania; ^26^ Institute of Marine Biology National Academy of Sciences of Ukraine Odessa Ukraine; ^27^ Department of Biological and Environmental Science University of Jyväskylä Jyväskylä Finland; ^28^ Fondation Hëllef fir d'Natur ‐ natur & ëmwelt Marnach Luxembourg; ^29^ Chair of Hydrobiology and Fisheries, Centre for Limnology Estonian University of Life Sciences Elva Estonia; ^30^ Department of Lake Fisheries National Inland Fisheries Research Institute Olsztyn Poland; ^31^ Biotechnical Faculty University of Ljubljana Ljubljana Slovenia; ^32^ CITAB‐UTAD ‐ Centre for Research and Technology of Agro‐Environment and Biological Sciences, Forestry Department University of Trás‐os‐Montes and Alto Douro Vila Real Portugal; ^33^ CIBIO, Centro de Investigação em Biodiversidade e Recursos Genéticos, InBIO Laboratório Associado, Campus de Vairão Universidade do Porto Vairão Portugal; ^34^ The Swedish Anglers Association Göteborg Sweden

**Keywords:** die‐off, disease, drought, ecosystem management, mussel, pollution, population decline, contaminación, declinación poblacional, enfermedad, manejo del ecosistema, mejillón, muerte masiva, sequía, 死亡, 贻贝, 疾病, 干旱, 污染, 种群数量下降, 生态系统管理

## Abstract

Mass mortality events (MMEs) are decimating populations and compromising key ecosystem functions around the globe. One taxon particularly vulnerable to MMEs is freshwater bivalve mollusks. This group has important ecosystem engineering capacities and includes highly threatened and highly invasive taxa. Thus, MMEs of freshwater bivalves have important implications for conservation and ecosystems. Despite this, little is known about the magnitude, frequency, duration, distribution, and causes of freshwater bivalve MMEs. Using a questionnaire, we compiled data from 239 reports describing freshwater bivalve MMEs across 22 European countries since 1960. With these data, we analyzed trends in MME timing, location, and magnitude; identified the species affected; and evaluated the suggested causes (including reporter certainty). We found that the frequency of reports of MMEs increased each year, MMEs affected a broad range of species, clear geographical patterns linking certain causes to specific locations were lacking, factors related to drying and habitat destruction predominated suggested causes, and considerable uncertainty surrounded the causes of many MMEs, particularly those associated with potential pollutants and disease agents. Based on our findings, we recommend the standardization of many aspects of MME research (e.g., reporting and recovery assessment protocols), increased surveying for MMEs, further investigation into the causes of MMEs, especially those with significant uncertainty, and immediate actions to improve waterbody management, mitigate the effects of high temperatures, and further protect freshwater bivalves through the development and implementation of appropriate management actions and legislation.

## Abstract (es)

Distribución, escala y causantes de los eventos de mortalidad masiva de los bivalvos de agua dulce en Europa

**Resumen:**

Los eventos de mortalidad masiva (EMM) diezman poblaciones y comprometen las funciones clave de los ecosistemas en todo el mundo. Un taxón particularmente vulnerable a los EMM es el de los moluscos bivalvos de agua dulce. Este grupo tiene capacidades importantes de ingeniería ecosistémica e incluye taxones altamente amenazados e invasivos. Por lo tanto, los EMM de los bivalvos de agua dulce tienen implicaciones importantes para la conservación y los ecosistemas. A pesar de ello, se sabe poco sobre la magnitud, la frecuencia, la duración, la distribución y las causas de estos EMM. Usamos un cuestionario para recopilar datos de 239 informes que describen los EMM de bivalvos de agua dulce en 22 países europeos desde 1960. Con estos datos, analizamos las tendencias en cuanto al momento, la ubicación y la magnitud de los EMM; identificamos las especies afectadas; y evaluamos las causas sugeridas (incluida la certeza de los informantes). Descubrimos que la frecuencia de los informes sobre EMM aumentaba cada año, que los EMM afectaban a una amplia gama de especies, que no existían patrones geográficos claros que relacionaran determinadas causas con lugares específicos, que los factores relacionados con la sequía y la destrucción del hábitat predominaban entre las causas sugeridas y que existía una considerable incertidumbre en torno a las causas de muchos EMM, en particular las asociadas a posibles contaminantes y agentes patógenos. Con base en nuestros hallazgos, recomendamos la estandarización de muchos aspectos de la investigación sobre EMM (por ejemplo, los protocolos de notificación y evaluación de la recuperación), el aumento de los estudios sobre EMM, una mayor investigación de las causas de los EMM, especialmente aquellos con un grado significativo de incertidumbre y la adopción de medidas inmediatas para mejorar la gestión de las masas de agua, mitigar los efectos de las altas temperaturas y proteger aún más los bivalvos de agua dulce mediante el desarrollo y la aplicación de medidas de gestión y legislación adecuadas.

## 欧洲淡水双壳类动物大规模死亡事件的分布、规模和驱动因素

**【摘要】** 大规模死亡事件(mass mortality event, MME)正在导致全球各地的生物种群数量锐减, 并破坏关键生态系统的功能, 而淡水双壳类软体动物是特别容易受到MME影响的类群之一。淡水双壳类具有重要的生态系统工程能力, 且包含高度濒危和高度入侵的类群。因此, 淡水双壳类的MME对保护和生态系统具有重要意义。尽管如此, 人们对淡水双壳类MME发生的严重程度、频率、持续时间、分布和原因仍知之甚少。本研究利用问卷调查, 整理了自1960年以来22个欧洲国家239份描述淡水双壳类MME报告的数据。我们利用这些数据分析了MME的发生时间、地点和严重程度的趋势, 确定了受影响的物种, 并评估了报告人提出的可能原因(包括报告人的确定性程度)。我们发现, MME的报告频率逐年增加, MME影响的物种范围广泛, 目前仍缺乏联系某些原因与特定地点的明确地理格局, 而与干燥和栖息地破坏有关的因素在报告人提出的可能原因中占主导地位, 许多MME的发生原因存在很大的不确定性, 尤其是与潜在污染物和疾病媒介有关的原因。根据以上结果, 我们建议对MME研究的多个方面进行标准化(如报告方法和恢复评估方法), 增加对MME的调查, 进一步调查MME的原因, 特别是那些具有较大不确定性的原因;此外, 还需立即采取行动来改进水体管理, 减缓高温的影响, 并通过制定和实施适当的管理措施和法律来进一步保护淡水双壳类动物。**【翻译:胡怡思;审校:聂永刚】**

## INTRODUCTION

Mass mortality events (MMEs) are large population losses due to substantial mortality occurring over a short time interval relative to the organism's generation interval (Fey et al., 2015). MMEs can have far‐reaching consequences, from altering food web structures, community compositions, and ecosystem functions (Baruzzi et al., 2018; Fey et al., 2019) to influencing the survival of species (García‐March et al., 2020). Recently studied MMEs include sea star wasting disease leading to mortality rates of up to 99–100% in *Pycnopodia helianthoides* populations across the Pacific coast of North America (Hamilton et al., 2021), the loss of 200,000 antelopes (*Saiga tatarica tatarica*) in 3 weeks due to the bacterium *Pasteurella multocida* type B (Kock et al., 2018), tsunami‐induced burial and starvation of the long‐lived and sparsely populated clam *Mercenaria stimpsoni* in Funakoshi Bay (Kubota et al., 2021), and the near extirpation of the now critically endangered fan mussel (*Pinna nobilis*) across the Mediterranean due to the interaction of multiple factors, including the protozoan parasite *Haplosporidium pinnae* and concurrent polymicrobial infections (Carella et al., 2023; Cinar et al., 2021; Kersting et al., 2019; Özalp & Kersting, 2020). MMEs are not necessarily confined to a single species; rather, they can affect multiple taxa simultaneously. This has been exemplified by numerous recent MMEs involving birds and mammals, driven by the rapid global spread of the highly pathogenic H5N1 avian influenza (Harvey et al., 2023; Leguia et al., 2023; Lycett et al., 2019).

MMEs may be a particular concern for species of high conservation need that are vulnerable to changes in environmental conditions due to a lack of mobility or play an important role in affecting wider community composition. One such taxon often affected by MMEs is freshwater bivalves, a diverse group spanning multiple orders and families (Downing et al., 2010; McDowell & Sousa, 2019; Neves, 1987; Waller & Cope, 2019). Freshwater bivalves are ecosystem engineers, providing many ecosystem functions, such as water filtration, enhancing habitat complexity, and changing nutrient cycling (Sousa et al., 2009; Zieritz et al., 2022). Thus, their loss could trigger cascading effects throughout the ecosystem (DuBose et al., 2019; Ollard et al., 2024; Sousa et al., 2012). The freshwater bivalves affected by MMEs include Unionida—a highly diverse and globally imperiled order consisting of 6 families and 1033 species (Böhm et al., 2021; Lopes‐Lima et al., 2018; MolluscaBase, 2025); highly successful invasive taxa, such as species in the Corbiculidae and Dreissenidae families (Veneroida and Myida, respectively) (Prestes et al., 2024); and extremely understudied but ecologically important species, such as the pea clams (Sphaeriidae, Sphaeriida) (Halabowski et al., 2024). Freshwater bivalves are highly sensitive to global change (Aldridge et al., 2023; Cushway et al., 2025), which stresses the need to understand the drivers and impacts of MMEs in this group.

A recent example of a freshwater bivalve MME was the loss of 50–90% of pheasantshell (*Actinonaias pectorosa* [Unionidae]) at different sites in the Clinch River (USA) due to recurring MMEs each year from 2016 to 2019 (Da Silva Neto et al., 2024; Leis et al., 2019; Richard, 2018; Richard et al., 2021, 2020). At one monitoring site, approximately 80,000 individuals (85.4%) were lost in a river stretch of just 200 m (Richard, 2018). Another example involves freshwater pearl mussels (*Margaritifera margaritifera* [Margaritiferidae]) in multiple rivers across Sweden, where mortality rates increased and reached up to 100% from 2011 to 2017 (Alfjorden et al., 2024; Wengström et al., 2019). The causes of these MMEs remain unknown, but they likely involved pathogens (Alfjorden et al., 2024; Da Silva Neto et al., 2024; Richard et al., 2021, 2020; Wengström et al., 2019).

Despite their ecological significance and imperiled status, freshwater bivalves are often overlooked because of their lack of economic value and perceived charisma (Mammola et al., 2023), resulting in a scarcity of information on the extent and causes of freshwater bivalve MMEs. The most in‐depth synthesis of information available today regarding freshwater bivalve MMEs comes from a workshop held in the United States in 1987, which described many large MMEs involving thousands of bivalves in North America that had mortality rates up to 99%. Despite these devastating numbers, little information was available about potential causes, with most cases being described as having unknown causes (Neves, 1987). Little has changed since then in understanding of the distribution, frequency, and drivers of MMEs in freshwater bivalves, despite their ongoing global declines (Aldridge et al., 2023). Although researchers may have hypotheses regarding the drivers of many MMEs, considerable uncertainty remains (Neves, 1987; Waller & Cope, 2019). We hypothesized that in some instances, even MMEs reported with a proposed cause are more accurately classified as resulting from unknown causes if the reporter provided their level of confidence in the assigned cause. Additionally, we hypothesized that ambiguous and cryptic causes, such as disease or pollution, are attributable to MMEs with lower confidence than more clearly identifiable causes, such as drying or flooding.

Considering the conservation value and threatened status of many freshwater bivalves, it is imperative to improve understanding of their MMEs; inform the development of appropriate scientific, societal, and policy measures to mitigate any anthropogenic factors contributing to their MMEs; and pinpoint areas for future research (Aldridge et al., 2023; Cossey et al., 2025; Waller & Cope, 2019). To address this need, we systematically collated a database of 239 MME reports of freshwater bivalves across Europe from 1960 to 2023. Using this database, we described existing knowledge on European freshwater bivalve MMEs, including their distribution, scale, and causes. We considered the conservation implications of these MMEs, including the essential next steps that must be taken in their study and effective management.

## METHODS

We sent a questionnaire (Appendix S1) to representative freshwater bivalve researchers from 34 countries across the geographical region of Europe who were involved in the CONFREMUS COST project (CA18239 ‐ Conservation of freshwater mussels: a pan‐European approach).

The questionnaire asked each representative to gather data on various aspects of MMEs in their country—including duration, distribution, magnitude, frequency, and causes—by engaging their national network of freshwater bivalve researchers.

All statistical analyses were carried out in R (R Core Team, 2024). Appendix S2 contains the final curated database (reported data and standardizations of data).

### Taxa affected, magnitude, and seasonality

Due to the nature of the reports, magnitudes of the MMEs were split into categories consisting of tens, hundreds, thousands, tens of thousands, hundreds of thousands, or millions of dead bivalves. Chi‐square goodness‐of‐fit tests were used to determine the dominant magnitude category and the most likely season of MMEs. Fisher's exact tests were used to analyze the relationship between the magnitude category of the event and both the taxa affected and the seasonality. Bonferroni adjustments were used to account for multiple comparisons.

### Causes, changes through time, duration, geographical distribution, and recovery

Changes in the frequency of MME reports each year over time were not analyzed with a formal statistical test due to inherent bias in these data (e.g., data availability and changes in effort over time, see “DISCUSSION”). Similar causes of MMEs were grouped into categories. The cause drying included reports with drought, low water, and/or desiccation listed as causes. The cause other hydrological changes included causes such as salinity fluctuations or sedimentation. The cause pollution encompassed chemical and biological pollutants (e.g., pesticides or algal biotoxins, respectively). There were no cases where physical pollutants (e.g., microplastics) were reported as a suggested cause. Chi‐square tests with Bonferroni adjustments to account for multiple comparisons were used to determine the dominant causes. Generalized linear models (GLMs), with quasi‐binomial distributions to account for overdispersion and logit link functions, were used to analyze changes in the proportion of MMEs reported with each of certain causes (those with more than 10 data points since 2010) over time (year). Only reports since 2010 were used when analyzing changes in the proportion of events attributed to specific causes to reduce the reporting bias present due to a general lack of study of bivalve MMEs before 2010. Fisher's exact test was used to detect any associations between magnitude category and cause. A Kruskal–Wallis test with Dunn's test and a Bonferroni correction for post hoc analyses was used to determine if cause had an impact on duration. A chi‐square goodness‐of‐fit test with Bonferroni correction was used to assess the chance of recovery.

### Assessing confidence in attributed causes

Considering that causes for MMEs may be suggested with varying levels of certainty, we included specific questions in the data collation questionnaire to categorize the confidence a reporter placed in their attributed cause (Appendix S1). Each answer was assigned a score (justifications in Appendix S1), and these scores were multiplied to calculate a confidence score ranging from 0 to 1. The confidence scores for each cause were compared using a Kruskal–Wallis test with Dunn's test and a Bonferroni correction for post hoc analyses. In many cases, there was low certainty in the attributed cause. To account for this uncertainty, we reclassified causes as unknown based on 5 thresholds of confidence scores (Appendix S1).

## RESULTS

### Taxa affected, magnitude, and seasonality

**FIGURE 1:**
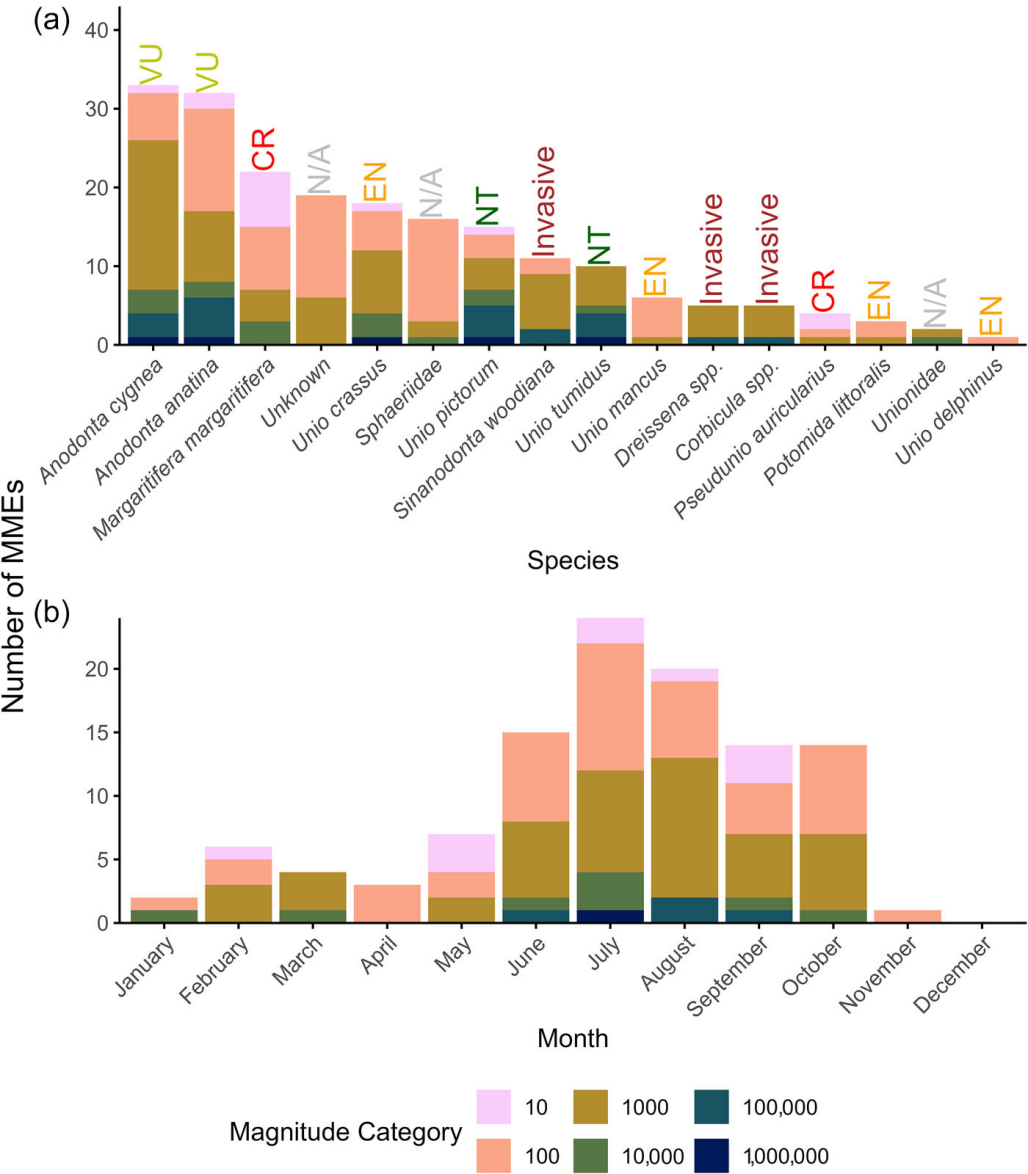
Number of freshwater bivalve mass mortality event (MME) reports in Europe of various magnitude categories (from tens of dead individuals to millions of dead individuals) by (a) species and (b) month from January to December (International Union for Conservation of Nature Red List categories: NT, near threatened; VU, vulnerable; EN, endangered; CR, critically endangered; N/A, not applicable; data from: https://www.iucnredlist.org/, accessed 26 February 2025). Range on the *y*‐axis differs between (a) and (b) because one MME may have affected multiple species.

A total of 239 MMEs were reported across 22 countries (examples shown in Appendix S3). Responses were obtained from 24 countries (71% of the countries that were sent the questionnaire). Two of these responses (Bosnia and Herzegovina and Italy) reported no MMEs. Reports involved at least 14 species of freshwater bivalves, spanning multiple orders (Figure 1a). Native and non‐native species were affected, including those assessed as vulnerable, endangered, and critically endangered by the IUCN Red List. The species with the third highest number of reports (22) was the freshwater pearl mussel (*M. margaritifera*), which is critically endangered in Europe. Of 127 reports with magnitudes listed, 99 (78%) were in the hundreds or thousands magnitude categories for the number of individuals killed. These categories were significantly more likely than all other magnitude categories (all χ^2^ *p* < 0.001). There were significant differences in the proportions of reports across magnitude categories among different taxa (*p* < 0.050, Fisher's exact), particularly for Sphaeriidae, which were often reported with lower magnitude categories than other taxa (Appendix S4).

MMEs were significantly more likely to be reported over the spring and summer months (April–September) than the autumn and winter months (October–March) (χ^2^_1_ = 47.761, *p* < 0.001) (Figure 1b). There was no difference in the proportion of events reported in each magnitude category for different months of the event (*p* = 0.863, Fisher's exact) (Figure 1b).

### Causes, changes through time, duration, geographical distribution, and recovery

**FIGURE 2:**
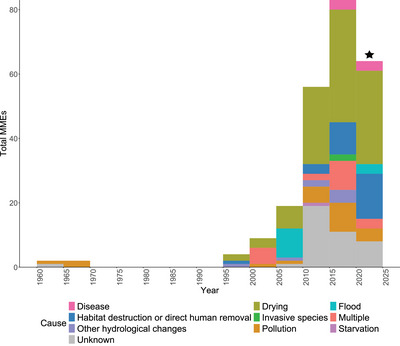
The number of freshwater bivalve mass mortality events (MMEs) in Europe reported within 5‐year blocks, grouped by the reporter's suggested cause (black star, final incomplete 5‐year category, which includes reports from 2020 to 2023 only).

Certain causes were more commonly reported as drivers of MMEs (Figure 2). Of interest, MMEs due to drying were significantly more likely to be reported than MMEs due to all other causes (41.8% of MMEs, all χ^2^ *p* < 0.005). The next most frequent cause was unknown (16.7% of causes). These reports were significantly more likely than reports with all remaining causes except habitat destruction or direct human removal, pollution, or multiple, which occurred at 11.7%, 9.6%, and 7.9%, respectively (all χ^2^ *p* < 0.005). There were no reports attributing MMEs solely to high temperatures. Disease was listed infrequently as a cause (only 6 reports; 2.5%).

The overall frequency of MME reports each year increased from 1960 to 2023 (Figure 2), with very few reports until 1995 and a subsequent rapid explosion of reports in the 2000s. There was an increase in the proportion of events attributed to habitat destruction or direct human removal since 2010 (*t*_211_ = 3.352, *p* = 0.001, GLM, quasi‐binomial distribution, logit link function). There was a significant decrease in the proportion of events with unknown causes since 2010 (*t*_211_ = −3.004, *p* = 0.003, GLM, quasi‐binomial distribution, logit link function). There were no significant changes in the proportions of events attributed to pollution, drying, or multiple causes since 2010 (all *p* > 0.050, GLM, quasi‐binomial distribution, logit link function). There was no difference in the proportion of events reported in each magnitude category for different causes (*p* = 0.088, Fisher's exact).

**FIGURE 3:**
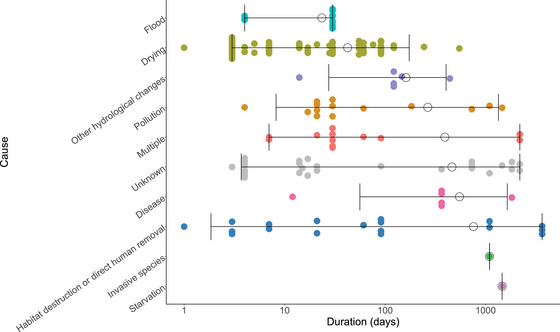
Duration (log scale) of freshwater bivalve mass mortality events in Europe by reporter's suggested cause (horizontal lines, 95% confidence interval; empty circles, mean; data points, offset from each other on the *y‐*axis).

MMEs were reported with a wide range of durations, ranging from a single day to 3650 days. There was no significant difference between causes in the duration of the MMEs when adjusting for multiple comparisons (all *H p* > 0.050) (Figure 3).

**FIGURE 4:**
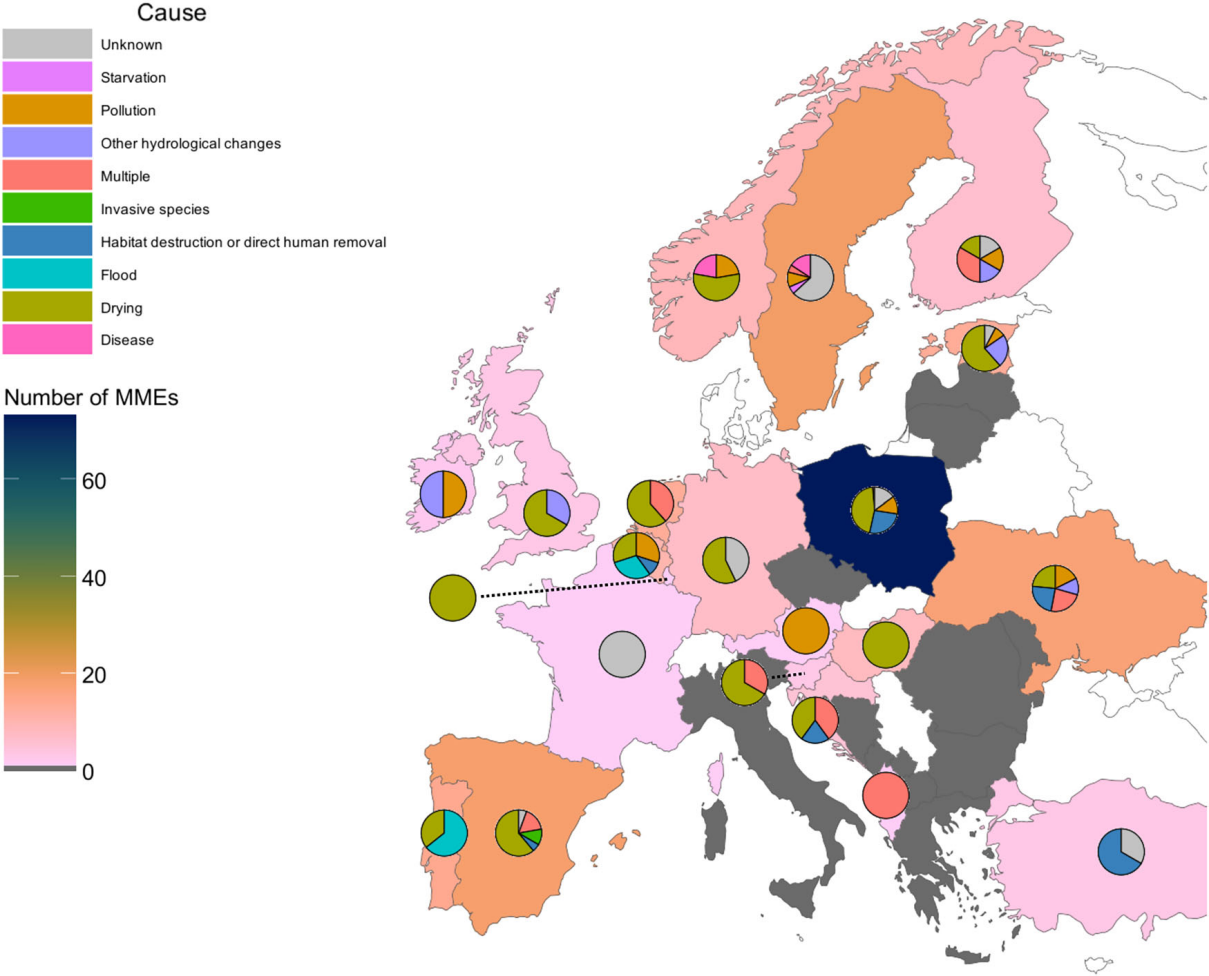
Distribution of freshwater bivalve mass mortality event (MME) reports in Europe, proportion of events attributed to each cause for each country (pie charts), and number of events reported in that country (gray, countries that did not respond to the request for information or that reported no MMEs [Bosnia and Herzegovina and Italy]; white, countries not included in the study).

MMEs were reported across Europe due to various causes (Figure 4). Poland had the largest number of MME reports (73). Sweden, Spain, and Ukraine also had many MME reports (19, 18, and 17 events, respectively). There were no latitudinal patterns in the reporting of MMEs. There were no systematic spatial trends in the distribution of reported causes across Europe. For example, MMEs with drying and flood as reported causes occurred in northern and southern countries.

Of the MME reports that suggested multiple causes, the interaction between pollution and other stressors was the most common (Appendix S5). MMEs with high temperature reported as a cause occurred only with either drying, low oxygen, or pollution as interacting causes.

Considering only reports before 2020 to allow time for recovery to be assessed, MMEs were significantly more likely to have no data available to assess recovery (117 events) than to have recovery observed (10 events) or recovery not observed (21 events) (both χ^2^ *p* < 0.001). The greater number of unrecovered compared with recovered populations was not significant (χ^2^_1_ = 3.903, *p* = 0.145).

### Assessing confidence in attributed causes

**FIGURE 5:**
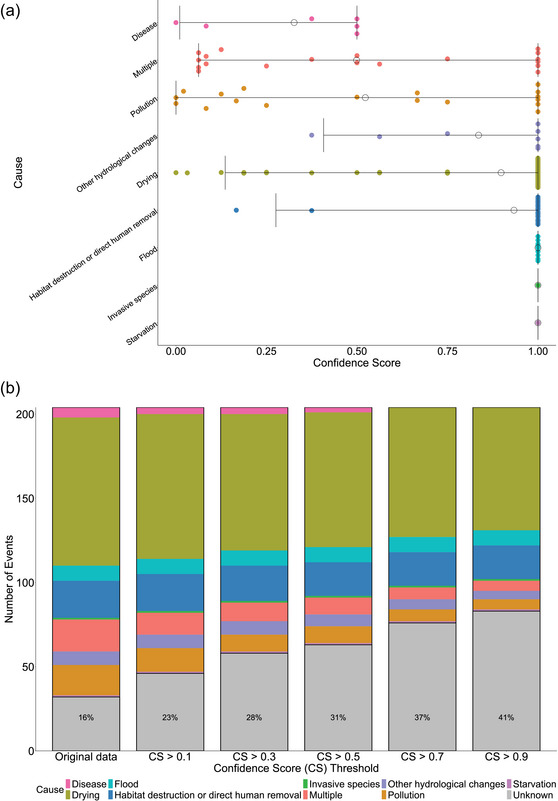
(a) Distribution of the confidence reporters had in the cause they attributed to freshwater bivalve mass mortality events in Europe (confidence score, product of the scores received for the answers given to a questionnaire, where each answer received a score from 0 to 1 [the higher the score, the greater the confidence] [details in Appendix S1]; horizontal lines, 95% confidence intervals; empty circles, mean; data points, offset from each other on the *y*‐axis for visualization) and (b) proportion of reports classified as unknown (gray) relative to all other causes when using the classifications provided in the original data and when reclassifying reports as unknown based on whether their confidence score (CS) was below a certain threshold (confidence score [CS] threshold).

There were significant differences in the confidence attributed to certain causes (*H*_7_ = 57.546, *p* < 0.005) (Figure 5a). Reporters were more confident when attributing drying, flood, or habitat destruction or direct human removal as the cause of an MME than disease, multiple, or pollution (all *H p* < 0.050). Approximately 16% of events were reported with unknown causes (Figure 5b). This percentage increased to 41% when the threshold above which a confidence score for a reported cause had to reach to be considered to have a known cause (confidence score threshold) was raised from 0 to 0.9. With a confidence score threshold of 0.5, the percentage of reports with unknown causes was 31% (Figure 5b).

## DISCUSSION

Our results showed that MMEs affected a wide range of species of varying conservation status, sizes, habitat types, and abundance. A larger number of MMEs were reported in the warmer months than the cooler months, and MMEs were primarily reported as killing hundreds or thousands of bivalves. The results also showed that there has been an increase in MME reports each year since 1960. Of reports with causes attributed to them, the 3 most dominant causes reported were drying, habitat destruction or direct human removal, and pollution. Our results also showed that MMEs were reported across a range of durations for all causes and that there was no clear geographical pattern in their distribution by cause. Pollution was the most common cause reported in association with other drivers.

The results confirmed our hypotheses that cryptically acting causes, such as disease and pollution, have, on average, lower confidence scores than more clearly acting causes, such as drying, habitat destruction or direct human removal, and flood. The confidence in reported causes also showed that there may be more MMEs with causes that are not well understood (up to 41% of reports) than suggested by the number of events initially reported as unknown.

### Taxa affected, magnitude, and seasonality

The larger number of MME reports in certain species could be attributable to differences in abundance, visibility, range, or susceptibility to MMEs among species. For example, *Anodonta cygnea* (Unionidae) is an abundant species in habitats (e.g., urban lakes) with a greater chance for encounter with the general public than rarer species that typically occur at low densities and are largely riverine (e.g., *Pseudanodonta complanata* [Unionidae]). This could partially explain the relatively large number of events reported for *A. cygnea* and other similarly distributed species. As expected, widely studied species, such as *M. margaritifera*, had more MME reports than relatively rare understudied species (e.g., *Pseudunio auricularius* [Margaritiferidae]) (Nakamura et al., 2023). MME reports were not limited to common species; *M. margaritifera* and *Potomida littoralis* (Unionidae) are critically endangered and endangered in Europe, respectively (Lopes‐Lima & Prié, 2024; Moorkens, 2024). There may be additional MMEs occurring in other understudied and highly threatened species that we are unaware of, such as the critically endangered *Unio tumidiformis* (Unionidae) and the endangered depressed river mussel (*P. complanata*), for which no events were reported.

The greater number of reports between spring and summer compared with autumn and winter months could be due to the corresponding increase in survey activity during the warmer period, meaning MMEs are more likely to be detected. Alternatively, more freshwater bivalve MMEs may occur in warmer months because the most common cause of MMEs in our database—drying events, a significant threat to bivalves globally (Cushway et al., 2025)—would be expected to occur more often in summer. Moreover, high temperatures, identified as a key interacting factor in both our study and in MMEs in marine bivalves (Soon & Ransangan, 2019), may contribute to an increased frequency of MMEs during warmer months.

The prevalence of MMEs in the hundreds and thousands magnitude range was expected because small MMEs are hard to detect and larger ones are difficult to quantify. The lack of magnitude patterns in MMEs across different factors (year, species, cause, time of year) likely reflected data limitations rather than ecological significance. Most reports lacked detailed quantification (e.g., “thousands of shells”), making statistical analyses of MME magnitude changes difficult. Therefore, our findings neither refute nor support previous studies showing particular trends, such as increasing MME magnitudes reported over time (Fey et al., 2015). The higher proportion of reports of Sphaeriidae MMEs in lower magnitude categories compared with many other taxa was surprising considering the potentially high density of Sphaeriidae populations, which can reach, for example, up to 130,000 individuals per square meter (Dyduch‐Falniowska, 1982; Halabowski et al., 2024). This may reflect a lack of quantification and recording of Sphaeriidae MMEs, in line with the general neglect of Sphaeriidae in freshwater ecological research and monitoring (Halabowski et al., 2024).

### Causes, changes through time, duration, and geographical distribution

The paucity of MME reports until 1995 and the subsequent explosion of reports in the last 2 decades could be driven by multiple factors. These patterns may reflect a true increase in MMEs due to an increased prevalence of stressors. Alternatively, the patterns may be a result of biases, such as an increased interest in researching MMEs and bivalves, or an artifact of the data collection, which required recall of the respondents. Even so, the pattern held for the last 15 years, which is within the career of most participants. The apparent drop in the number of reports in the 5‐year category from 2020 to 2025 is likely due to the incomplete nature of this category. In addition, this category included the COVID‐19 years, during which the study of MMEs would have been hindered.

Despite the importance of disease in driving mortality events in other taxa (Carella et al., 2023; Fey et al., 2015; Hamilton et al., 2021; Hewson et al., 2025; Sanderson & Alexander, 2020; Tracy et al., 2019), very few reports in our database attributed MMEs to disease. This may be because of a lack of knowledge about diseases in freshwater bivalves or because diseases are more difficult to study than other causes, such as drying events. Indeed, reporters were, on average, less confident in attributing an MME to disease than to other causes.

Pollution was another frequently reported MME cause, both alone and in combination with other interacting causes. Freshwater bivalves are particularly sensitive to pollutants; indeed, pollution is a major threat driving bivalve declines globally (Cope et al., 2021; Downing et al., 2010; Haag, 2012). Despite this, details on what specific pollutants were involved in the MMEs in our database were limited and reporters’ confidence in attributing pollution as a cause was low, potentially due to the difficulty of establishing the causative role of pollutants in bivalve declines (e.g., Woolnough et al., 2020). In general, little is known about the sensitivity of European freshwater bivalves to pollutants, and very few studies exist in Europe on the subject (Belamy et al., 2022; Nakamura et al., 2021).

It is difficult to know whether the range of durations reported for MMEs is a true phenomenon or an artifact of data collection. It could be that reporters checked a site once every few years and noticed the disappearance of a bivalve population. Over such long periods, it may be better to classify such cases as slower declines rather than MMEs, unless additional (unreported in our database) reasons existed to conclude that the loss of the bivalves occurred over a small time window within the sampling years. This potential misclassification of durations and of events as MMEs may contribute to the lack of an observed relationship between the cause of an MME and its duration. Despite this lack of significant effect, the general trends suggested that certain causes, such as floods, may act more quickly than others, such as diseases, as might be expected.

Although there were no clear geographical patterns in the possible causes of MMEs, there were certain countries, such as Poland, with a higher frequency of reports than others, such as the United Kingdom. Differences in the number of MME reports between countries could be due to a variety of factors, including differences in true MME prevalence, country size, research activity, habitat accessibility, or reporting efforts. The process of studying bivalves varies drastically by region in Europe, and public and media engagement with conservation varies across the continent, meaning that encounter rates and frequency of reporting MMEs may be contributing to these patterns.

Of the countries without MME reports, some reported a lack of MMEs (Bosnia and Herzegovina and Italy), whereas others did not respond to requests for data—despite, in some cases, having a strong contingent of freshwater bivalve researchers. This could be due to a lack of MMEs in these countries, a lack of MME expertise, or a lack of interest or time to participate in the study.

### Ecological and conservation importance

The insights gained from our results enhance understanding of freshwater bivalve MMEs. This is essential due to the highly endangered status of many species (Böhm et al., 2021; Lopes‐Lima et al., 2018), the ecosystem services they provide (Vaughn, 2018; Zieritz et al., 2022), and the wider ecosystem alterations that can occur due to freshwater bivalve MMEs (Bódis, Tóth, & Sousa, 2014; DuBose et al., 2019; McDowell et al., 2017).

The loss of freshwater bivalves during MMEs could lead to a loss of habitat structure for other organisms, especially if shells decay (Bódis, Tóth, Szekeres, et al., 2014; Ollard, 2024). Considering that freshwater bivalves are filter feeders, massive decreases in bivalve numbers during MMEs can lead to decreases in water quality due to a loss of biofiltration (Vaughn et al., 2015).

In instances where an MME results in a large biomass of bivalves rapidly dying, the event can extensively alter the direct freshwater ecosystem and the adjacent terrestrial ecosystems (Novais et al., 2017, 2015). In the short term, decomposing bivalve carrion provides a nutrient pulse that could stimulate food web productivity (Bódis, Tóth, & Sousa, 2014; DuBose et al., 2019; Fey et al., 2019; McDowell & Sousa, 2019; McDowell et al., 2017). In cases where floods cause MMEs, bivalves can be stranded on waterbody banks when waters recede, causing a nutrient pulse into terrestrial systems (Sousa et al., 2012). The availability of bivalve carrion can cause aggregative responses and increased reproductive rates of consumers. In turn, this can act as a resource pulse for higher trophic‐level consumers, thus stimulating changes in multiple trophic levels (Fey et al., 2019; Novais et al., 2015; Sousa et al., 2012; Yang et al., 2008). Bivalve carrion can enter the detrital food web, which could stimulate changes in microbial communities and nutrient cycling, driving further bottom‐up changes to food webs (Benbow et al., 2020; Yang, 2004). Long‐term alterations to nutrient cycles are possible after MMEs of freshwater bivalves as shells continue to release nutrients into the system for many years (Ilarri et al., 2019, 2015; McDowell & Sousa, 2019) and decreased biofiltration rates reduce the exchange of nutrients between the water column and the benthos (Baustian et al., 2014; DuBose et al., 2019; Vaughn et al., 2015).

### Future directions and management actions

**FIGURE 6:**
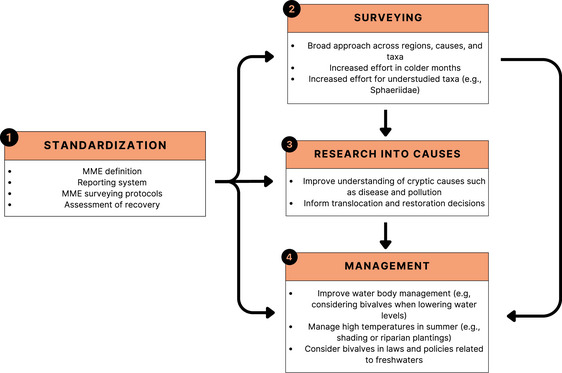
Freshwater bivalve mass mortality event (MME) future research directions and management actions (arrows, how each step feeds into the next). Although knowledge growth progresses according to the flowchart, leading to more informed management, actions should be taken simultaneously in all areas where possible. For example, the management actions provided could be immediately implemented while the standardization of terms, increased surveying, and more research into causes will work to inform additional key management actions for the future. Examples given for each subset are not exhaustive.

Considering the consequences of bivalve MMEs for conservation and the wider functioning of freshwater and terrestrial ecosystems, it is surprising that so little is known about their distribution, scale, and causes. Without a more structured understanding of the patterns, causes, and consequences of MMEs, effective management actions cannot be implemented. We suggest that there are steps to be taken to fill important knowledge gaps necessary for more effective management in the future (Figure 6, boxes 1–3). These steps include standardization of MME reporting and surveying, broader surveys for MMEs across taxa, and increased research effort into cryptically acting causes, such as disease and pollution. We also recommend management actions be taken with the knowledge available now, including the consideration of bivalves in waterway management, alleviation of high‐temperature effects, and increased recognition of bivalves in freshwater laws and policies (Figure 6, box 4).

Our data and their limitations highlight the need for the development of standardized methods for sufficiently defining and reporting MMEs to facilitate easier analysis. There is currently no widely accepted, standardized definition of what constitutes an MME (e.g., the required number of affected individuals or duration of the event). The highly variable durations of reported MMEs demonstrate the need for the development and application of such a standardized definition, with a reporting framework that can capture the timescale and magnitude of the event. Without this, various phenomena may be reported as MMEs, including slower declines. These different phenomena may require different considerations for appropriate management and response (e.g., the speed of action and resource allocation). A standardized reporting framework would also allow for key data from MMEs to be integrated into existing threat assessment frameworks (e.g., the IUCN Red List) (IUCN Standards and Petitions Committee, 2024). Such a reporting framework need not be restricted to freshwater bivalves but could be applied to the reporting of MMEs across many taxa, possibly in the form of an easily accessible app. This would facilitate a greater understanding of the conservation impacts of MMEs for a population or species, informing potential management actions, such as the need for captive breeding programs (Geist et al., 2023).

Standard procedures for regularly surveying bivalve populations should be developed and applied. To date, there is only one European Committee for Standardisation (CEN) standard for freshwater mussel monitoring available, and it focuses on the freshwater pearl mussel (Boon et al., 2019). Procedures for surveying an MME upon its discovery should also be standardized and made accessible. For example, when collecting data on bivalve MMEs, it would also be informative to report whether MMEs happened concurrently in other taxa. Although some advice exists about surveying and sampling aquatic MMEs (Abila et al., 2018; Cossey et al., 2025; Marchowski et al., 2024; Meyer & Barclay, 1990; Southwick & Loftus, 2017; Work, 2015), a standard survey regime for other taxa that may not be the initial focus of an MME investigation should be established. Such standardization would reduce uncertainty regarding whether a report mentioning only bivalves reflects a true specificity to bivalves or a lack of adequate sampling effort for other taxa and improve understanding of the causes and ecological impacts of MMEs.

In addition, some MMEs were reported without information regarding the species or genus affected. If an MME is observed by a nonspecialist who cannot identify the affected organism, collecting shells or taking photographs for later identification should be encouraged. It is difficult to properly manage and respond to MMEs when information as basic as what organisms are affected is unknown. This is especially important for difficult‐to‐identify taxa, such as Sphaeriidae, for which no reports identified the affected genus or species.

The paucity of information given in MME reports regarding recovery emphasizes the need for standardized, systematic, and justified methods for assessing the recovery of populations post MMEs, akin to those that exist for assessing fish stocks (Cotter, 2007; Hammer et al., 2010; ICES, 2022a, 2022b, 2024; Powers, 2003). Standardized recovery assessment reporting would facilitate a greater understanding of the factors associated with recovery, provide important context around the severity of different MMEs, and inform future management actions.

Bivalve populations should be regularly surveyed using standardized methodologies to allow for the accurate identification of population trends and MMEs (Dobler et al., 2024; Ollard & Aldridge, 2023). Considering the widespread nature of MMEs of various causes across many species and locations in Europe, a broad approach that is not restricted to a few common species, limited habitats, or certain causes in specific locations is necessary. Increased effort surveying in the colder months should be undertaken to determine whether the increase in MME reports over warmer months is of ecological relevance or a sampling artifact. Greater surveying for MMEs in understudied, rare, and highly threatened taxa is needed. This is especially important for Sphaeriidae, a taxon that had limited data in our database, is often overlooked, and yet is of high ecological importance (Halabowski et al., 2024).

The decrease in the proportion of events with unknown causes since 2010 may reflect the increasing research interest in the ecology and conservation of freshwater bivalves over the period but may also result from an increased prevalence of MMEs driven by readily attributable factors, such as habitat disturbance. Despite a decrease in the proportion of MMEs with unknown causes, reporters still had much uncertainty regarding causes, especially cryptically acting ones, underscoring the need to invest more resources into research on MME causes. It seems reasonable to assume that cryptically acting causes, such as disease or pollution, may contribute to many MMEs with no known causes (Brian & Aldridge, 2023; Cope et al., 2021; Waller & Cope, 2019). Focusing efforts on understanding the effects of pollution and diseases on freshwater bivalves may engender a better path toward successful MME management. For example, it is difficult to make management decisions for MMEs that may be caused by pollution or pathogens without knowledge of what specific pollutants or pathogens are acting as causative agents. As a first step, future research should apply recent guidelines to improve the sampling and study of MMEs and bivalve diseases (Cossey et al., 2025; Knowles et al., 2023; Waller & Cope, 2019). Specifically, local biologists and conservation workers should be trained and prepared to rapidly sample and preserve materials from an MME before degradation leaves only shells (Cossey et al., 2025). This is imperative considering the rapidly acting nature of many MMEs.

Further research into the causes of MMEs with a specific focus on informing future bivalve translocations is also needed. Captive breeding of freshwater bivalves as a conservation tool to augment wild populations is widespread across Europe (Geist et al., 2023), with new technologies for housing and breeding bivalves, especially freshwater mussels, being developed and tested (Douda et al., 2021; Lima et al., 2012; Rock, 2024). However, this presents an important problem: the risk of spreading pathogenic agents through translocations of bivalves to and from breeding facilities and wild populations for restoration efforts (Brian et al., 2021). Although there is much information available about the metazoan parasites of freshwater bivalves (reviewed in Brian & Aldridge [2019] and Grizzle & Brunner [2009]), studies on the freshwater bivalve microbial pathogens that may be linked to MMEs are limited (Da Silva Neto et al., 2024; Leis et al., 2023; Richard et al., 2021, 2020, 2022), especially in Europe (Alfjorden et al., 2024). It is essential to build knowledge around what potential pathogens may be common to MMEs in Europe and their etiological roles to facilitate the development of procedures, such as rapid screening tests, that can be applied to bivalves before translocations.

Future management actions should be informed by the knowledge gained through the steps outlined above (Figure 6, boxes 1–3). However, some actions can be taken now, informed by the results of this study (Figure 6, box 4). The dominance of MMEs reported with causes such as drying and habitat destruction or direct human removal presents an opportunity for the easy mitigation of many future MMEs by improved management of freshwater ecosystems. Many of the reports in these 2 categories were due to controlled human activities, such as water level lowering for construction activities, dredging, draining for fishing, or dam management. These MMEs, due to avoidable mismanagement, affected species across a broad range of conservation statuses, including vulnerable, endangered, and critically endangered species. They could have been avoided by accounting for the potential impact on bivalves of waterway management activities. Considering that the frequency of reported MMEs caused by habitat destruction and direct human removal has been increasing since 2010, simply improving management around this issue could help avoid many future MMEs.

It would be valuable to increase efforts in managing stressors to bivalve populations over warmer months during which they may be more susceptible to certain drivers of MMEs. In addition, the mitigation of high temperatures should be considered in future management plans to avoid its additive impact with other stressors and the potential for water body drying. For example, the shading of waterways known to harbor bivalve populations could be considered, such as by increasing riparian plantings, which are known to be beneficial for freshwater bivalve populations (Popov et al., 2025).

Given the highly threatened status of many European freshwater bivalves and the frequent MMEs affecting them, it is essential that current and future climate, biodiversity, and environmental legislation consider their conservation (IUCN SSC MSG/CPSG/CONFREMU, 2024). Moreover, the development of additional regulations at the regional, national, or European level should be explored to better protect these understudied and undervalued taxa. A greater inclusion of freshwater bivalves in law—and the enforcement of these laws—would facilitate the uptake of the management actions and future directions we have highlighted.

By collating and analyzing 239 MME reports from the last 63 years, we have shed light on MMEs in freshwater bivalves, a group containing important and globally imperiled taxa. Based on our results, we identified a need for the standardization of many aspects of MME research; increased surveying of MMEs across a broad range of geographies; further investigation into cryptic MME drivers, such as disease and pollution; and immediate action to protect freshwater bivalves through appropriate management and legislation. Our findings are not only of relevance to freshwater bivalves but should be applied more broadly, especially considering the recent increases in the frequency and magnitude of MME reports across many taxa (Fey et al., 2015).

## Supporting information




**Supplementary Material**: cobi70192‐sup‐0001‐AppendixS1.docx

**Supplementary Material**: cobi70192‐sup‐0002‐AppendixS2.xlsx

**Supplementary Material**: cobi70192‐sup‐0003‐AppendixS3.docx

**Supplementary Material**: cobi70192‐sup‐0004‐AppendixS4.docx

**Supplementary Material**: cobi70192‐sup‐0005‐AppendixS5.docx
