# Supplementary material for: Distribution, scale, and drivers of mass mortality events in Europe's freshwater bivalves
Source: Conserv Biol. 2025 Dec 18;40(2):e70192. doi: 10.1111/cobi.70192 (PMC13036312; doi:10.1111/cobi.70192)
Supplement: Supplementary file 1 — Supplementary Material: cobi70192‐sup‐0001‐AppendixS1.docx [file COBI-40-e70192-s004.docx]

appendix s1: Questionnaires

A questionnaire in the form of an Excel spreadsheet was sent to each participating researcher to gather information about MME reports (Questionnaire Part A). In addition to this questionnaire, a multiple-choice questionnaire designed to assess the confidence a reporter had in their suggested cause for the MME was also sent to each participant (Questionnaire Part B). Both questionnaires were distributed in English; however, all participants had the means to understand or translate the questions if necessary. Despite this, reading the questionnaires in a second language may have contributed some additional variability to the results, particularly for Questionnaire Part B. The answers to both questionnaires were collated into a final database (these data, along with our standardisations, can be found in Appendix S2).

The contents of the questions in Questionnaire Part B were adapted from previous work (Christie et al., 2023; White et al., 2022) for the specific case of guiding a participant in assessing their confidence in the evidence behind their claims. In this case, the aim was to assess the level of confidence that should be placed in their claim about what caused a particular MME. Questionnaire part B included a series of hypothetical examples to help the participant decide into which category the evidence behind their suggested cause would fall. The hypothetical examples are intended to give general guidance on what falls into each category. They may be interpreted with some degree of subjectivity and are not intended to be an exhaustive list of all evidence types that would fall into each category. Thus, the results of the questionnaire were interpreted with caution. The questionnaire could be easily adapted to assess confidence in other claims.

The four questions of Questionnaire Part B were designed to address two primary aspects of the confidence of the reporter in their suggested cause; namely, the reporter’s confidence that the suggested cause was capable of killing mussels, assuming it happened, and the confidence of the reporter that the suggested cause actually happened (Table 1., column 3). Each question had four potential answers (Categories 0 – 3), each with a score between 0 and 1 (Table 1). The scores received for each of the four questions were multiplied to calculate the confidence score used in further statistical analyses. Question 2 was graded differently (Table 1) because, unlike all other questions, if Category 0 (no general evidence that the suggested cause can cause mortality) was selected, we deemed that the total confidence in the cause should also be 0, irrespective of other responses. These questions and answers were distributed as a Google Form to participants.

*Table 1. The questions used in Questionnaire Part B, what they contributed to addressing, and the scoring system used for the multiple-choice answers (each potential answer to a question is a category).*

| Question number | Question | What the question addressed | Scoring |
| --- | --- | --- | --- |
| 1 | How reliable is the source of your evidence for your suggested cause? | The confidence the reporter had that the suggested cause was involved in the specific mortality event. | Category 0 received 1/4 points, Category 1 received 2/4 points, Category 2 received 3/4 points, and Category 3 received 4/4 points. |
| 2 | How reliable is the evidence that links your suggested cause with mussel MMEs in general? | The confidence the reporter had that the suggested cause would kill mussels, assuming it happened. | Category 0 received 0 points, Category 1 received 1/3 points, Category 2 received 2/3 points, and Category 3 received 3/3 points. |
| 3 | How similar are the conditions of the general evidence (explored in the previous question) to the conditions of the specific MME you have reported? | The confidence the reporter had that the suggested cause would kill mussels, assuming it happened. | Category 0 received 1/4 points, Category 1 received 2/4 points, Category 2 received 3/4 points, and Category 3 received 4/4 points. |
| 4 | How certain are you that your suggested cause was the cause of this specific MME? Tick the option that best describes your situation. | The confidence the reporter had that the suggested cause was involved in the specific mortality event. | Category 0 received 1/4 points, Category 1 received 2/4 points, Category 2 received 3/4 points, and Category 3 received 4/4 points. |

Without appropriate validation data, it was impossible to determine the exact relationship between the confidence score and the likelihood that the suggested cause was the true cause of the MME. To address this while still illustrating the potential increase in MMEs without known causes when factoring in reporter confidence, we applied five confidence score thresholds (0.1, 0.3, 0.5, 0.7, 0.9) to recategorise MMEs as having known or unknown causes.

We expect that the format we have used to assess the confidence of reporters in their suggested causes could be generalised and easily applied to other cases when a rapid assessment of confidence in a claim (from an external party or your own claim) is needed. It may be useful to translate the questionnaire into the native language of participants in future applications.

Questionnaire Part A.

*Cells sent in an Excel spreadsheet to each researcher to gather information about MME reports.*

| **Information about the MME requested** |
| --- |
| Researcher name |
| Country |
| Date |
| Duration |
| Location |
| Latitude [ddd.ddd] |
| Longitude [ddd.ddd] |
| Water body type |
| Water body type if "other" |
| Name of the water body (geographic or local/common if any exists) |
| Waterbody characteristics |
| Trophic state |
| Additional information/remarks (e.g. protected area, recreation area, other ecosystems impacted) |
| Type of water reservoir |
| Average depth [m] |
| Maximum depth [m] |
| Extent of the kill (range of the die-off incident) - stream/river length [km]; lake area [m^2^] |
| Mussel species affected |
| Other mussel species present in the site |
| Were only mussels affected? |
| Were fish killed, too? |
| Other biota affected: algae, macrophytes, zooplankton, insects, crayfish, vertebrates |
| Quantity of dead mussels |
| Shell examination |
| Internal examination |
| Mussel population data (e.g. density, size, age and sex structure of the dead mussels vs. live ones) |
| Water temperature in the reservoir during the incident [degrees Celsius] |
| Organization/entity investigating the incident |
| Measurements taken (e.g. pH, dissolved oxygen, conductivity, temperature, NH_4_^-^N, NO_3_-NO, NO_2_-NO, TN, TKN, phosphates, sulfides, total organic carbon, BOD5, COD, total suspended solids, hydrogen sulphide or other (please specify)) |
| Actions or measures deployed to minimise or compensate effects |
| Reasons for mussel death and potential impact sources (e.g., pollution, human activities, natural disaster, discharge pipe, runoff ditch, habitat disruption, others (please specify) and unknown) |
| Countermeasures undertaken to eliminate such incidents and protect mussels in the future |
| Possible recovery signs discovered or any trends of the mussel population affected |
| Types of the information sources (e.g. scientific literature, grey paper, media report, personal observation or other (please specify)) |
| Details about information sources (web links, paper references, databases/datasets, etc.) |
| Comments and remarks |
| Answers to confidence score questionnaire (below) |

Questionnaire Part B.

*Multiple-choice questionnaire to assess the confidence of a reporter in their suggested cause*

1) How reliable is the source of your evidence for your suggested cause? Tick the option that best describes your situation. If you are the sole source of the information, please evaluate yourself as a source. In this question, biases include, but are not limited to, a need to present knowledge when it is lacking, a desire to slander the name of another group/individual, or a personal monetary investment in the issue.

| Category 0, no source: The source is lost, or you can no longer remember the source. |
| --- |
| Category 1, unreliable source: Option 1 - people with little connection to or knowledge of freshwater mussels or the area; Option 2 - official reports of bodies/individuals known to have a prior interest that might introduce biases.  For example:  A friend from out of town who is not involved with freshwater mussels or the area of interest.  An industry representative not willing to expose poor pollution management. |
| Category 2, partially reliable source: Option 1 - people with general knowledge about freshwater mussels but not specific knowledge about the MME; Option 2 - locals with no connection to freshwater mussels; Option 3 - reports that are not-peer reviewed but come from groups with no known biases.  For example:  A local resident of the area who had never seen a freshwater mussel before noticing them dead after an MME.  An unbiased newspaper report. |
| Category 3, reliable source: Option 1 - peer-reviewed scientific paper or official government report; Option 2 – local resident with a relevant connection to freshwater mussels and the specific area where the MME occurred; Option 3 – you observe the MME. In all cases, potential biases of the source should be deemed as minimal.  For example:  A local angler with many years of experience living around the water body.  A government report not produced for the public (to avoid potential bias towards providing a “known” cause). |

2) How reliable is the evidence that links your suggested cause with mussel MMEs in general? General evidence refers to evidence from the wider world (not the specific MME) about the suggested cause and its link to mortality (Salafsky et al., 2022). Please tick the most appropriate option.

Please note that 1) when these options refer to correlations and experiments, these do not have to be undertaken/observed by scientists but could be based on local knowledge and 2) when these options refer to reliability, this indicates considerations such as the inclusion of necessary controls, the potential for biases, the use of appropriate measurement methods, and the inclusion of adequate sample numbers.

| Category 0, no general evidence: You have a hypothesis that your suggested cause can lead to mortality, but it has never been observed or experimentally studied before and is based solely on speculation. Alternatively, the cause is completely unknown.  For example: A pollutant that has never been studied before but might kill mussels. |
| --- |
| Category 1, weak general evidence: few reports of mortality that are not reliable.  For example:  A single study that experimentally infected mussels with a suspected disease-causing agent but did not include any controls.  One report of “high” temperature in a stream immediately before an MME. There are either no historical records of temperature to provide a baseline and/or the temperature was measured unreliably (e.g., someone felt the water seemed slightly warmer than normal when swimming). |
| Category 2, medium strength general evidence: Option 1 - a small number of highly reliable reports of mortality; Option 2 - many reports of mortality that are not very reliable.  For example:  Many studies that experimentally infected mussels with the suspected disease-causing agents undertaken in unreliable ways, e.g., without controls and with a small sample size.  A single report of an experimental infection with the suspected disease-causing agent undertaken reliably, e.g., with non-infected mussels for a procedural control and with an appropriate sample size.  One report of a certain contaminant in a stream at elevated levels immediately before an MME compared to historical records of this contaminant to provide a baseline. The contaminant was measured reliably. |
| Category 3, strong general evidence: many reports of mortality that are highly reliable.  For example:  It has been observed in many cases that increases in temperature compared to background control temperatures, as recorded on temperature loggers, have directly preceded MMEs.  Many studies that experimentally infected mussels with suspected disease-causing agents undertaken in reliable ways, e.g., with controls and with an appropriate sample size. |

3) How similar are the conditions of the general evidence (explored in the previous question) to the conditions of the specific MME you have reported?

| Category 0, no similarity: None of the conditions match between the general evidence for the suggested cause and the specific MME or there is no evidence for the cause. Alternatively, the cause is completely unknown. |
| --- |
| Category 1, weak similarity*:* The evidence that your suggested cause can lead to mortality was gathered at a significantly different time (e.g., different season); and place (e.g., different climate or habitat type, such as tropics vs arctic); and using an unrelated species to the one impacted during the MME.  For example:  The evidence that a pollutant observed during an MME of freshwater mussels in the dry season in the tropics is responsible for the MME comes from a field experiment of mortality of marine bivalves in the near arctic during the winter storm season.  The evidence that low dissolved oxygen during an MME of freshwater mussels in the dry season in the tropics is responsible for the MME comes from observations of anoxic conditions causing mortality of marine bivalves in the near arctic during the winter storm season. |
| Category 2, medium similarity*:* The evidence that your suggested cause can lead to mortality fulfils one or more, but not all, of the following conditions: Condition 1 – the evidence was gathered at a similar time to the MME (e.g., season); Condition 2 - the evidence was gathered at a similar place (e.g., climate or habitat type) to the MME; or Condition 3 - the evidence was gathered using similar species to the MME.  For example:  The evidence that the pollutant observed during an MME of freshwater mussels in the dry season in a tropical river is responsible for the MME comes from a field experiment on the mortality of freshwater mussels in an arctic lake during the winter season.  The evidence that low dissolved oxygen during an MME of freshwater mussels in the dry season in the tropics is responsible for the MME comes from observations of anoxic conditions causing mortality of freshwater crayfish in the tropics during the dry season. |
| Category 3, high similarity*:* The evidence that your suggested cause can lead to mortality was gathered at a similar time (e.g., same season); and a similar place (e.g., same climate or habitat type, such as both from the tropics); and using a similar species to the MME.  For example:  The evidence that the pollutant observed during an MME of freshwater mussels in the dry season in the tropics caused the MME comes from a field experiment on the mortality of freshwater mussels in the tropics during the dry season.  The evidence that low dissolved oxygen during an MME of freshwater mussels in the dry season in the tropics is responsible for the MME comes from observations of anoxic conditions causing mortality of freshwater mussels in the tropics during the dry season. |

4) How certain are you that your suggested cause was the cause of this specific MME? Tick the option that best describes your situation.

| Category 0, no evidence: No observed evidence that your suggested cause was relevant to the specific MME reported. Alternatively, the cause is completely unknown.  For example:  Previous MMEs in your country were associated with increased temperature, but there is no connection between temperature and this specific MME.  It is well known that climate change is associated with mortality but there is no evidence that climate change factors were associated with this specific MME. |
| --- |
| Category 1, low certainty: There is some evidence that your suggested cause occurred at approximately the right time and location to have caused the MME. Either the magnitude of your suggested cause is unknown or whether your suggested cause can kill sufficient mussels at the observed magnitude is unknown.  For example:  A farmer started adding pesticides of unknown toxicity to mussels onto crops right next to the site of the MME a few weeks before mortality was observed. |
| Category 2, medium certainty: There is good evidence that your suggested cause occurred at the right time and location to have caused the MME and some evidence that it occurred in approximately the right magnitude to have caused the MME where a mechanism of action linking your suggested cause and mussel mass mortality is known.  For example:  A farmer started adding pesticides known to be highly toxic to mussels onto crops. The pesticides were subsequently detected at roughly the right concentration (e.g., the LC_50_) to cause mortality at the location of the MME in both mussel tissues and the water during the event. |
| Category 3, high certainty: There is very strong evidence that your suggested cause occurred at the right time, location, and magnitude to have caused the MME with no uncertainty, where a mechanism of action linking your suggested cause and mussel mass mortality is known.  For example:  Records of the river drying up at the time of the MME and mussels left desiccated without water for long enough to cause mortality.  Records of dredging and leaving mussels to die on the shore or other direct human destruction to the mussels.  Clear evidence of predation, e.g., piles of dead mussel shells known to be from otter activity or human removal.  Experimentally exposing a group of healthy mussels to a classified disease agent or pollutant isolated from the waterbody of the MME caused similar mortality to the MME. |

References

Christie, A. P., Morgan, W. H., Salafsky, N., White, T. B., Irvine, R., Boenisch, N., Chiaravalloti, R. M., Kincaid, K., Rezaie, A. M., Yamashita, H., & Sutherland, W. J. (2023). Assessing diverse evidence to improve conservation decision-making. *Conservation Science and Practice*, *5*(10), e13024. https://doi.org/10.1111/csp2.13024

Salafsky, N., Irvine, R., Boshoven, J., Lucas, J., Prior, K., Bisaillon, J.-F., Graham, B., Harper, P., Laurin, A. Y., Lavers, A., Neufeld, L., & Margoluis, R. (2022). A practical approach to assessing existing evidence for specific conservation strategies. *Conservation Science and Practice*, *4*(4), e12654. https://doi.org/10.1111/csp2.12654

White, T. B., Amano, T., Boersch-Supan, P., Christie, A. P., Freckleton, R., C. Quinzin, M. C., Rezaie, A. M., Sutherland, W. J., & Yamashita, H. (2022). 2. Gathering and Assessing Pieces of Evidence. In W. J. Sutherland (Ed.), *Transforming Conservation* (1st ed., pp. 31–74). Open Book Publishers. https://doi.org/10.11647/OBP.0321.02
