# Supplementary material for: Distribution, scale, and drivers of mass mortality events in Europe's freshwater bivalves
Source: Conserv Biol. 2025 Dec 18;40(2):e70192. doi: 10.1111/cobi.70192 (PMC13036312; doi:10.1111/cobi.70192)
Supplement: Supplementary file 3 — Supplementary Material: cobi70192‐sup‐0003‐AppendixS3.docx [file COBI-40-e70192-s003.docx]

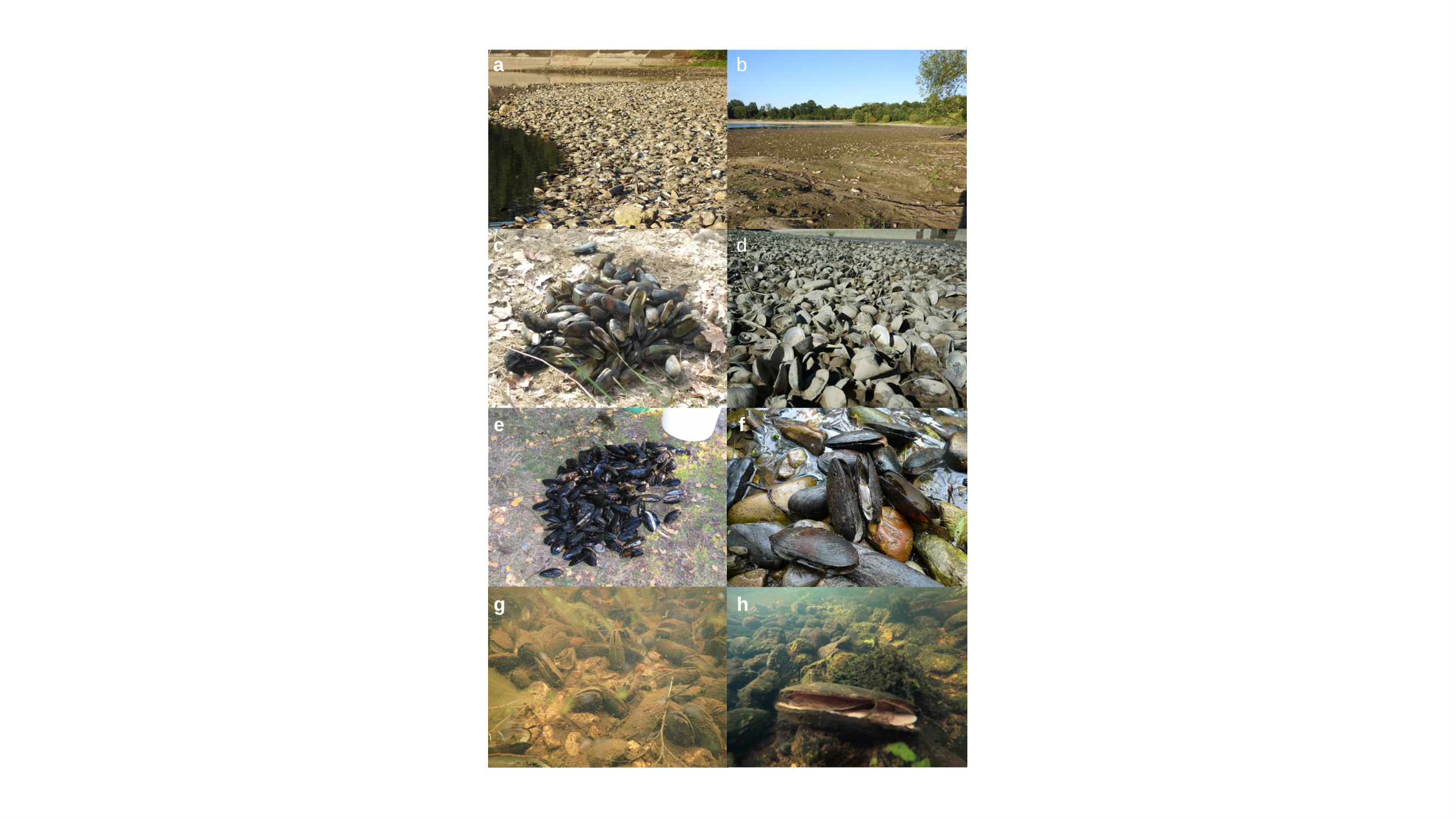


Appendix S3. Examples of freshwater bivalve mass mortality events (MMEs) in Europe: (a, b) swan mussels (*Anodonta cygnea*), duck mussels (*Anodonta anatina*), painter's mussels (*Unio pictorum*), and swollen river mussels (*Unio tumidus*) left to dry after water was drained from Witoszówka Reservoir, Poland, in 2020 (photos by Jarosław Słowikowski), (c) *Pseudunio auricularius* shells collected from an MME caused by several simultaneous events in the Canal Imperial de Aragón, Spain, in 2018 (photo by Keiko Nakamura), (d) *Corbicula fluminea* shells in the substrate of the Canal Imperial de Aragón, Spain, after the water level was lowered in 2012 (photo by Keiko Nakamura), (e) Freshwater pearl mussel (*Margaritifera margaritifera*) shells collected from a suspected pollution event in Harbe Aist River, Austria, in 2006 (Photo by blattfisch e.U.), (f) freshwater pearl mussels killed in the river Eo, Spain, because of changes in the river level during dam works in 2012 (Photo by the Fish and Molluscs Conservation Research Group, University of Santiago de Compostela), and (g, h) empty freshwater pearl mussel shells partially embedded in or resting on the surface of the substrate intermingled with intact and moribund individuals (g) and an example of a recently dead freshwater pearl mussel (h) from an MME suspected to have been caused by disease in Lakselva River, Norway, in 2023 (photos by Jon Magerøy and Daniel Cossey).
