# Supplementary material for: Distribution, scale, and drivers of mass mortality events in Europe's freshwater bivalves
Source: Conserv Biol. 2025 Dec 18;40(2):e70192. doi: 10.1111/cobi.70192 (PMC13036312; doi:10.1111/cobi.70192)
Supplement: Supplementary file 4 — Supplementary Material: cobi70192‐sup‐0004‐AppendixS4.docx [file COBI-40-e70192-s001.docx]

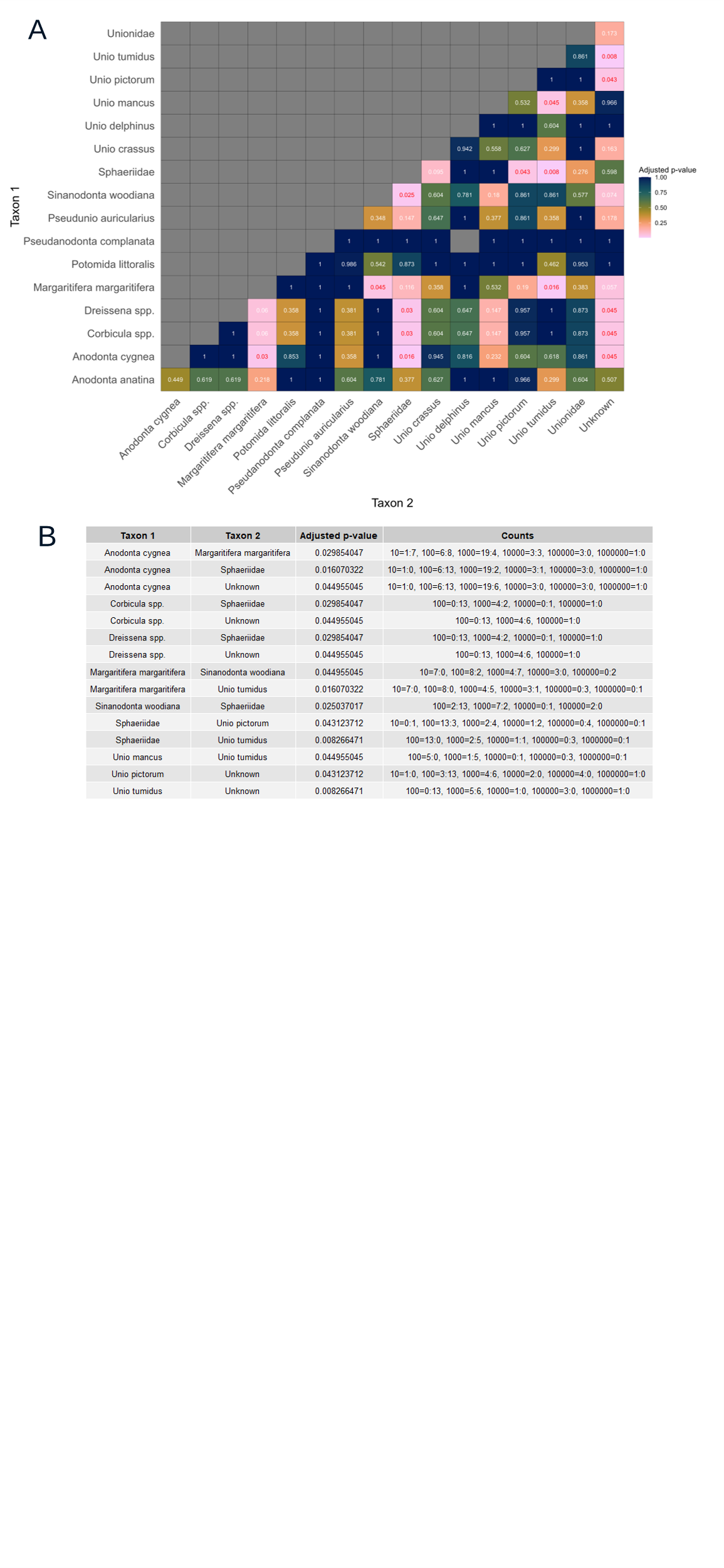


Appendix S4. Difference in the magnitude categories of reported MMEs between each taxon. A) The adjusted p-values (Benjamini-Hochberg corrections of Fisher’s exact tests) of each pairwise comparison of the different proportions of reports in each magnitude category between every taxon. B) The corresponding counts in each magnitude category for each of the comparisons displayed in A. The magnitude categories are tens (10), hundreds (100), thousands (1000), tens of thousands (10000), hundreds of thousands (100000), and millions (1000000). The counts for each taxon in each category are displayed as taxon 1: taxon 2.
