# Supplementary material for: Distribution, scale, and drivers of mass mortality events in Europe's freshwater bivalves
Source: Conserv Biol. 2025 Dec 18;40(2):e70192. doi: 10.1111/cobi.70192 (PMC13036312; doi:10.1111/cobi.70192)
Supplement: Supplementary file 5 — Supplementary Material: cobi70192‐sup‐0005‐AppendixS5.docx [file COBI-40-e70192-s005.docx]

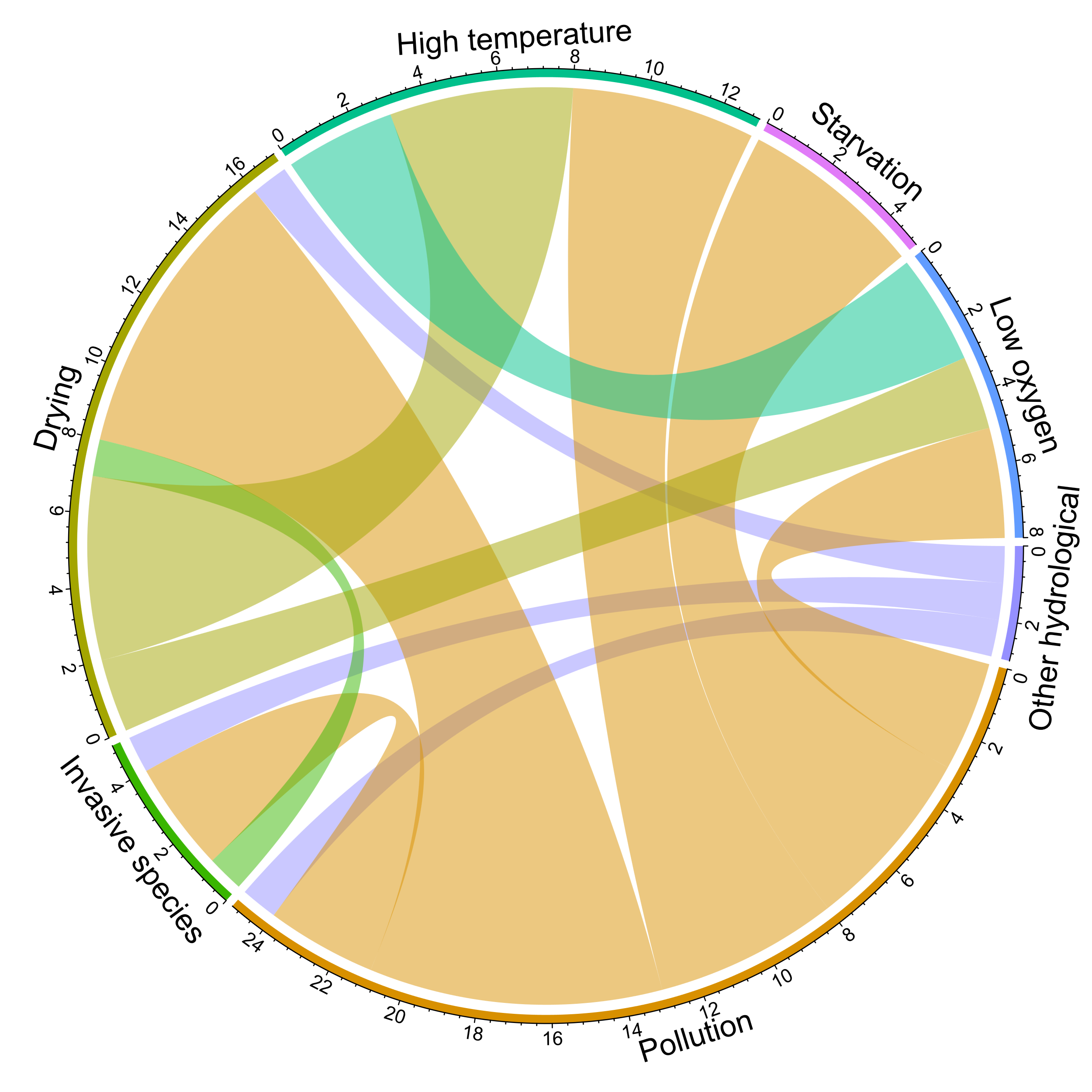
Appendix S5. Split of the causes suggested for freshwater bivalve mass mortality events (MMEs) in Europe when multiple causes were reported for a single MME (numbers, frequency of each pairwise interaction; connected causes, causes reported together).
